# Immunological and hemato-biochemical effects on catfish (*Clarias gariepinus*) exposed to dexamethasone

**DOI:** 10.3389/fphys.2022.1018795

**Published:** 2022-09-16

**Authors:** Alaa El-Din H. Sayed, Hesham Taher, Hamdy A. M. Soliman, Alaa El-Din Salah El-Din

**Affiliations:** ^1^ Zoology Department, Faculty of Science, Assiut University, Assiut, Egypt; ^2^ Department of Water Biology, Faculty of Fish and Fisheries Technology, Aswan University, Aswan, Egypt; ^3^ Department of Zoology, Faculty of Science, Sohag University, Sohag, Egypt; ^4^ Zoology Department, Faculty of Science, Aswan University, Aswan, Egypt

**Keywords:** pharmaceutical residue, blood biomarkers, antioxidant, cytokines, catfish

## Abstract

Dexamethasone (glucocorticoid) was recently shown to be a life-saving drug for the treatment of *SARS-CoV-2* disease. Water and sediments can be contaminated by sewage treatment plants when this product is widely used. Accordingly, we evaluated the effects of dexamethasone as pharmaceutical residue on *Clarias gariepinus*, following exposure and post-exposure recovery on blood biochemical, antioxidant, and cytokine markers. Three experimental groups were examined. Control, fish exposed to 0.3 mg/L of dexamethasone, and fish exposed to 3 mg/L of dexamethasone for 7 days, followed by a 15-days recovery period. Hematological indices, such as red blood cell number, hemoglobin (Hb), platelets, mean corpuscular hemoglobin concentration, and large lymphocytes, were significantly declined following the exposure to dexamethasone compared to control. In contrast, hematocrit (Ht), mean corpuscular volume, monocytes, small lymphocytes, and mean corpuscular hemoglobin increased significantly depending on the dose–concentration. Liver and kidney functions, other biochemical parameters (albumin and globulin), cortisol, and cytokine (IL-1β and IL-6) concentrations increased significantly after exposure to dexamethasone compared to control. Antioxidants and acetylcholinesterase enzymes were significantly decreased in catfish treated with dexamethasone cumulatively with doses. After a recovery period, blood biochemical, antioxidant, and cytokine markers were still elevated compared with the control group. In conclusion, dexamethasone at concentrations present in water bodies causes deleterious effects on blood biomarkers, biochemical, and antioxidant as well as immune upregulation in catfish until after depuration period.

## Introduction

Recently, human and veterinary pharmaceuticals are contaminating aquatic ecosystems (Soliman et al., 2019). Quantifying active pharmaceutical ingredients (APIs) has been accomplished by measuring surface water, ground water, effluents, and biota (Togola and Budzinski, 2008). Inflammation, autoimmune diseases, and cancer are among the conditions that are treated with glucocorticoids (Miyoshi et al., 1997). Dexamethasone has anti-inflammatory and immunosuppressive effects and is a powerful synthetic glucocorticoid drug. It is used to treat a wide range of illnesses, including allergies, asthma, COVID-19, rheumatic problems, and skin diseases (Miyoshi et al., 1997; Sanders et al., 2020). There have been high levels of trace amounts of dexamethasone detected in sewage effluent, which belongs to the glucocorticoid cortisone family (Herrero et al., 2013). Brank et al. (1998) reported that river water collected downstream from a French pharmaceutical factory contained high levels of dexamethasone (10 mg/L); however, the concentration in the sewage effluents was reported as 0.3 mg/L (Chang et al., 2007).

Fish health has become increasingly concerned as a result of aquatic pollutants (Sayed and Soliman, 2017). Environmental risk assessment for pharmaceutical contaminants is based on several approaches. Among such contaminants, DEXA is recognized for its detrimental effects on brown trout (Pickering et al., 1987). Dexamethasone has been reported to adversely affect fathead minnow growth, reproduction, and development (LaLone et al., 2012). It also inhibits hepatic CYP450 in juvenile rainbow trout (Carletti et al., 2007), causes oxidative stress and reduced testosterone concentrations in *Hopliasmala baricus* (Guiloski et al., 2015), affects the hematological and immunological response in freshwater fish (Ribas et al., 2016), and induces hepatomegaly and steatosis in larval stage of zebrafish (Haider and Rauf, 2014). The highest concentrations of dexamethasone have been found in wastewater from hospitals, suggesting that it will likely increase in different environmental matrixes due to its increase during the COVID-19 pandemic (Musee et al., 2021). Data on DEXA’s effects on marine aquatic ecosystems and wildlife are limited. For an improved management of dexamethasone, studies involving long-term toxicological outcomes, realistic exposure concentrations, and whole organisms are needed. As a result, human lives can be saved and ecological integrity can be protected.

African catfish (*Clarias gariepinus*) is a toxicological model and has been used in immunological and biomedical studies as an excellent model (Mekkawy et al., 2011). Accordingly, we investigated the effects of high and low-dose dexamethasone on this model using hematological, biochemical, antioxidants, and immunological biomarkers.

## Materials and methods

### Chemicals

Dexamethasone sodium was purchased from Sigma-Aldrich, United Kingdom.

### Fish collection

Juveniles of the African catfish, *C. gariepinus*, were collected from the aquaponic unit at Assiut University. Fish is fed twice (5% body weight) with commercial pellets daily. The conditions were as 27–28°C with a 12 h:12 h light–dark cycle in multiple tanks (24 fish/100 L each) for 2 weeks to acclimate before the experiments. A daily replacement of approximately 20% of water was performed during the acclimation period. Fish ranged between 23–34 cm in total length and 83–140 g in weight. They were examined before the experiments to determine whether the fish were healthy and parasite-free (Fish and Service, 2010).

### Dexamethasone exposure

Catfish are capable of absorbing drug molecules from water through their mucosal skin, gills, and gut (Peatman et al., 2015). Dexamethasone was dissolved in dimethyl sulphoxide (DMSO) solution, and the solutions were diluted in double-deionized water before use as 3–0.3 mg/L. Briefly, the selected concentration was chosen because it environmental relevant concentration (Brank et al., 1998), where a significant alteration in physiology was observed, but gross morphology and survival of the organisms were not affected. In addition, the dose of the current study ranged from 0.3–3.0 mg/L was selected according to Guiloski et al. (2015). The acclimatized fish (n = 8/group in triplicates) were randomly divided into three groups: control, exposed to 0.3 mg/L dexamethasone (low dose), and exposure to 3.0 mg/L dexamethasone (high dose) for 7 days followed by a 15-days recovery period. Food was supplied to each aquarium for 1 h before dosing to minimize the removal of dexamethasone from residual food and feces in the test water and water partially substituted every day. The water content of dissolved oxygen, pH, and temperature were 8.23 ± 4.5 mg/L, 6.7 ± 0.34, and 25 ± 3°C, respectively. After exposure, four fish randomly selected from each treatment and benumbed using ice (Hamed et al., 2019). Blood samples (1.5 ml) were collected from the caudal vein into heparinized tubes for the measurement of hematological, biochemical, antioxidants, and immunological indices, and the rest of fish remained in tanks without treating (recovery period) for 15 days. Experimental setup and fish handling were according to the guidelines of the Research and Ethical Committee of the Assiut University.

### Haemato-biochemical parameters

Hematological parameters, such as RBCs, WBCs, differential WBCs, blood platelets, HCT, and Hb were estimated using a hematocytometer and other related parameters such as MCV, MCHC, and MCH were calculated using equations according to Mekkawy et al. (2011).

Colorimetric estimation of the biochemical parameters in serum (creatinine, uric acid, urea, liver enzymes (AST and ALT), glucose, total protein, and albumin) were measured with a spectrophotometer (Jasco-V530) at wavelength (340–546 nm), depending on the parameter being tested, according to Hamed et al. (2019).

### Antioxidants and cortisol measurements

Based on the method described by Knedel and Böttger (1967), serum acetylcholinesterase (AchE) was measured using Stanbio kits. Cortisol was measured by enzyme-linked immunosorbent assay (ELISA) according to Foster and Dunn (1974). According to Habig et al. (1974), glutathione-s-transferase activity was determined. Superoxide dismutase (SOD) was measured according to Nishikimi et al. (1972). Total antioxidant capacity (TAC) was measured using kits (Sigma-Aldrich, United States). A thiobarbituric acid reaction was used to measure malondialdehyde (MDA) (Ohkawa et al., 1979).

### Immunological parameters

The serum cytokines, IL-1β and IL-6, were measured by ELISA kits using ultrasensitive commercial kits (Human Ultrasensitive, Biosource International Inc.), according to Wang and Secombes (2009) and Hanington and Belosevic (2007), respectively.

### Statistical analysis

Data were described by the mean of samples ±SE. A one-way analysis of variance was used to compute the results (SPSS software package, Version 15) (Stevens, 2013). Values of *p* < 0.05 were considered significant.

## Results

### Biochemical parameters

Kidney function (creatinine, urea, and uric acid) showed significant (*p* < 0.05) increases after exposure to 0.3–3.0 mg/L dexamethasone for 7 days in dose-dependent manner compared to control, while serum globulin and albumin levels show a significant decrease with the two treated groups compared with the control. The liver functions such as the aspartate aminotransferase (AST) level became higher in the high concentration group only. On the other hand, alanine aminotransferase (ALT), glucose, and total protein levels appeared with no significant change in the results (Table 1). After the recovery period, higher levels of the biochemical indices were still detected in the dexamethasone-exposed groups compared with the control group (Table 1).

**TABLE 1:** Biochemical parameters (means ± SE) of the African catfish (*Clarias gariepinus*) after dexamethasone exposure (7 days) and recovery (15 days).

	Exposure period			Recovery period		
	Control	Low	High	Control	Low	High
		Dexamethasone	Dexamethasone		Dexamethasone	Dexamethasone
Creatinine (mg/dl)	0.35 ± 0.01^a^	0.35 ± 0.0^a^	0.47 ± 0.0^b^	0.35 ± 0.0^a^	0.34 ± 0.0^b^	0.35 ± 0.0^a^
Uric acid (mg/dl)	2.3 ± 0.1^a^	2.5 ± 0.1^b^	2.5 ± 0.1^b^	2.2 ± 0.1^a^	2.3 ± 0.0^a^	2.6 ± 0.1^b^
Urea (mmol/l)	22.6 ± 0.4^a^	23.8 ± 0.1^ab^	24.3 ± 0.8^b^	22.6 ± 03^a^	23 ± 0.2^ab^	23.7 ± 0.1^b^
AST (µ/L)	34.1 ± 0.9^a^	34.1 ± 0.3^a^	36.6 ± 0.5^b^	33.7 ± 0.4^a^	34 ± 0.3^a^	34.6 ± 0.4^a^
ALT (µ/L)	17.4 ± 0.4^a^	17.6 ± 1^a^	19.8 ± 1.4^a^	17.7 ± 0.2^a^	17.8 ± 0.2^a^	18.7 ± 0.0^b^
Glucose (mg/dl)	73.7 ± 2.8^a^	82.4 ± 3.4^a^	85.3 ± 5.7^a^	76.8 ± 0.6^a^	77.3 ± 0.8^a^	84.4 ± 3.3^b^
Total protein (mg/dl)	4.1 ± 0.3^a^	4.2 ± 0.1^a^	4.3 ± 0^a^	3.8 ± 0.1^a^	3.7 ± 0.0^a^	4.2 ± 0^b^
Albumin (mg/dl)	1.7 ± 0.1^a^	1.5 ± 0.9^b^	1 ± 0^c^	1.8 ± 0.0^a^	1.7 ± 0.0^a^	1.5 ± 0^b^
Globulin (g/dl)	2.3 ± 0.3^a^	2.7 ± 0.1^b^	3.2 ± 0.1^b^	2.6 ± 0.0^a^	2 ± 0.0^b^	2 ± 0.0^c^

Values with different superscript letters for the same raw and single sampling time (exposure and recovery) are significantly different (*p* < 0.05).

### Hematological parameters

Hematological indices including erythrocyte count (RBCs) were significantly (*p* < 0.05) decreased after exposure to 0.3–3.0 mg/L of dexamethasone for 7 days compared with the control group. The Hb and MCH values were found to be higher at low dexamethasone concentration compared to high concentration and control (Table 2). In contrast, small lymphocytes were significantly (*p* < 0.05) increased after exposure to 0.3–3.0 mg/L of dexamethasone. Monocyte and eosinophil noted with a significant increase in values only in the high dose exposure while large lymphocyte, MCHC, and thrombocyte were opposite as their levels only marked with a significant decrease only in the higher concentration treated group for 7 days on the other side, white blood cells did not change after the exposure period (Table 2). After the recovery period, only neutrophil, monocyte, eosinophil, MCV, and Ht returned to the normal levels in the treated groups compared to the control group. Large lymphocyte and small lymphocyte still unchanged after the recovery period compared with the control. The other hematological indices were detected unchanged in levels in the high dose treated group compared with the control group (Table 2).

**TABLE 2:** Hematological profile (means ± SE) of the African catfish (*Clarias gariepinus*) after dexamethasone exposure (7 days) and recovery (15 days).

	Exposure period			Recovery period		
	Control	Low	High	Control	Low	High
		Dexamethasone	Dexamethasone		Dexamethasone	Dexamethasone
(RBC’s) (Million/mm^3^)	3.2 ± 0.04^a^	3.07 ± 0.03^ab^	3 ± 0.1^b^	3.1 ± 0.1^a^	3.1 ± 0.1^a^	2.8 ± 0^b^
Hemoglobin (Hb) (g/dl)	8.9 ± 0.14^a^	9.6 ± 0.1^b^	8.4 ± 0.2^c^	9.1 ± 0.1^a^	9.6 ± 0.1^b^	9.6 ± 0.1^b^
Ht (PCV) (%)	35.4 ± 0.1^a^	36.9 ± 0.2^b^	35.3 ± 0.1^a^	35.6 ± 0.1^a^	35.9 ± 0.6^a^	34.6 ± 0.3^a^
MCV (µm³)	115.7 ± 1.9^a^	119.9 ± 1.4^b^	117.7 ± 2.3^b^	115.7 ± 2.2^a^	116.7 ± 3.4^a^	122.6 ± 0.5^a^
MCH (Pg)	28.3 ± 0.3^a^	31.1 ± 0.3^b^	27.9 ± 0.5^a^	29.6 ± 0.7^a^	31.1 ± 0.9^a^	34.1 ± 0.2^b^
MCHC (%)	25.4 ± 0.5^a^	25.9 ± 0.2^a^	23.7 ± 0.5^b^	25.5 ± 0.2^a^	26.7 ± 0.6^ab^	27.8 ± 0.3^b^
Thrombocytes (thousands/mm^3^)	213 ± 0.5^a^	217 ± 2.9^a^	206 ± 1.3^b^	212 ± 0.9^a^	214 ± 0.9^a^	207 ± 1.4^b^
(WBC’s) (thousands/mm^3^)	10.9 ± 0.2^a^	10.8 ± 0.3^a^	10.5 ± 0.2^a^	11.3 ± 0.1^a^	11.2 ± 0.0^a^	10.6 ± 0.2^b^
Neutrophils (%)	10.5 ± 0.3^a^	11.3 ± 0.3^a^	13.5 ± 0.3^b^	10.5 ± 0.3^a^	11.3 ± 0.3^a^	11.5 ± 0.5^a^
Large lymphocyte (%)	72.8 ± 0.3^a^	71.3 ± 0.5^a^	60.8 ± 0.9^b^	72.8 ± 0.3^a^	71.3 ± 0.5^b^	71.3 ± 0.3^b^
Small Lymphocyte (%)	12 ± 0.4^a^	13.5 ± 0.3^b^	19 ± 0.6^c^	11.8 ± 0.3^a^	13.5 ± 0.3^b^	13 ± 0.0^b^
Monocyte (%)	2.8 ± 0.3^a^	2.8 ± 0.3^a^	3.8 ± 0.3^b^	3 ± 0.0^a^	2.8 ± 0.3^a^	2.8 ± 0.3^a^
Eosinophils (%)	2 ± 0^a^	2 ± 0^a^	2.8 ± 0.3^b^	2 ± 0^a^	2 ± 0^a^	2 ± 0^a^

Values with different superscript letters for the same raw and single sampling time (exposure and recovery) are significantly different (*p* < 0.05).

### Antioxidants and stress indices

The levels of total antioxidant capacity (TAC), superoxide dismutase (SOD), and acetylcholinesterase exhibited a significant decrease (*p* < 0.05) after exposure, especially to the high dose (3 mg/L) for 7 days compared with the control group, while a significant increase in levels were noted in cortisol and malondialdehyde after high dose, and there was no change in glutathione-s-transferase levels with the two treated groups (Table 3). After the recovery period, the activities of the tested antioxidant still with a significant difference in the high dose treated group compared with the control group except glutathione-s-transferase and cortisol levels (Table3).

**TABLE 3:** Antioxidant parameters (means ± SE) of the African catfish (*Clarias gariepinus*) after dexamethasone exposure (7 days) and recovery (15 days).

	Exposure period			Recovery period		
	Control	Low	High	Control	Low	High
		Dexamethasone	Dexamethasone		Dexamethasone	Dexamethasone
Acetylcholinesterase (µ/L)	553 ± 5^a^	538 ± 6^a^	439 ± 9^b^	540 ± 6^a^	540 ± 3^a^	452 ± 8^b^
Cortisol (µg/dl)	13.8 ± 0.7^a^	12.5 ± 0.1^a^	16.5 ± 0.7^b^	13.9 ± 0.9^a^	13.5 ± 0.4^a^	15.6 ± 0.8^a^
Glutathione-S-transferase (U/ml)	37.5 ± 2.8^a^	34.2 ± 1^a^	33.7 ± 0.3^a^	39 ± 2.6^a^	35.8 ± 0.5^a^	35.5 ± 0.4^a^
Superoxide dismutase (U/ml)	2.7 ± 0.1^a^	2.3 ± 0.1^b^	1.9 ± 0.1^b^	2.6 ± 0^a^	2.4 ± 0.1^ab^	2.2 ± 0^b^
Total antioxidant capacity (nmol/L)	57.6 ± 4.4^a^	44.2 ± 0.4^b^	43.1 ± 1.2^b^	56.5 ± 3^a^	45.3 ± 0.5^b^	44.7 ± 1^b^
Malondialdehyde (nmol/ml)	15.7 ± 1.3^a^	18.1 ± 0.4^a^	30.9 ± 2.6^b^	15.7 ± 0.9^a^	19.5 ± 0.4^b^	33.5 ± 0.4^c^

Values with different superscript letters for the same raw and single sampling time (exposure and recovery) are significantly different (*p* < 0.05).

### Immunological parameters

**FIGURE 1:**
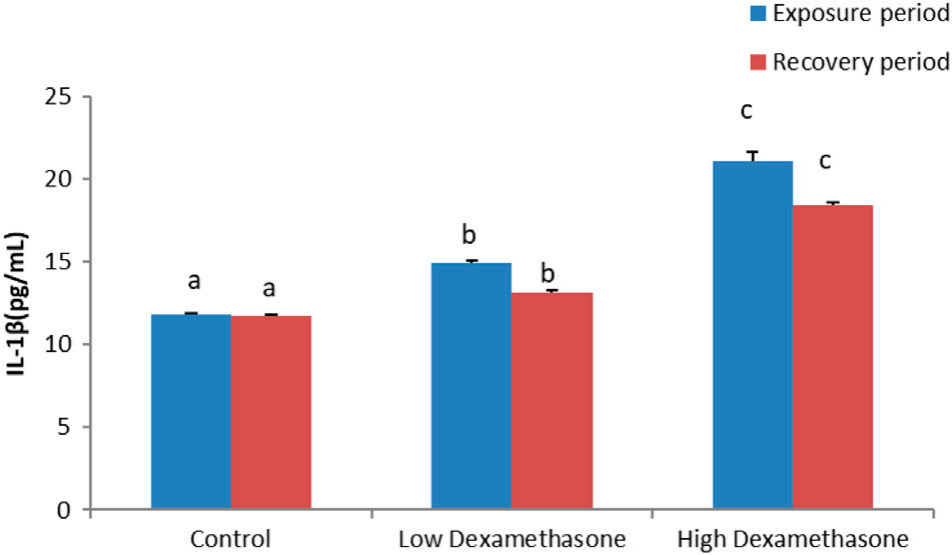
IL-1β concentration in the African catfish (*Clarias gariepinus*) after dexamethasone exposure (7 days) and recovery (15 days).

**FIGURE 2:**
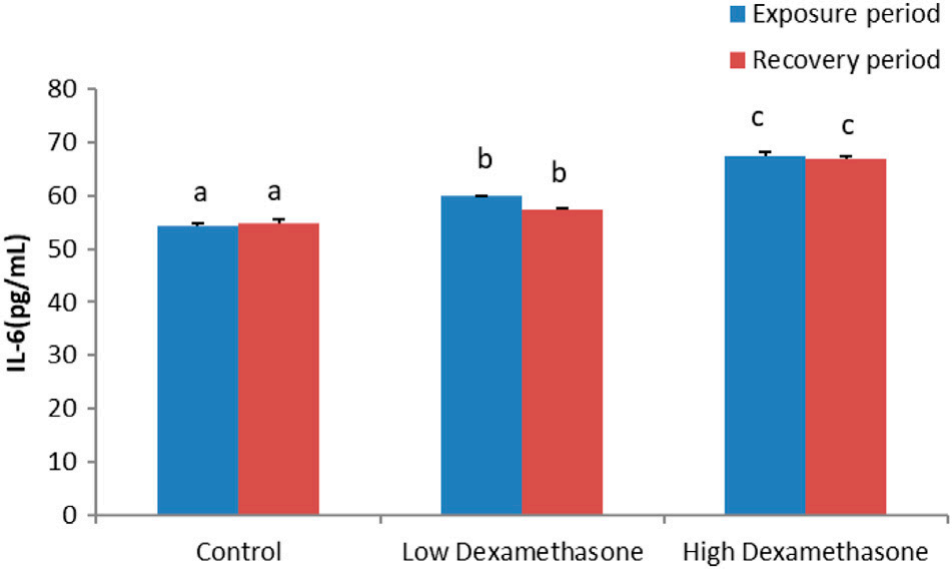
IL-6 concentration of the African catfish (*Clarias gariepinus*) after dexamethasone exposure (7 days) and recovery (15 days).

The cytokines, IL-1β and IL-6, exhibited a significant increase in the serum of the catfish after exposure to low and high doses of dexamethasone (Figures 1, 2). After the recovery period, the activities of the cytokines are still with a significant difference in the treated groups compared to the control group (Figures 1, 2).

## Discussion

There have been some studies describing the effects of synthetic glucocorticoids on aquatic vertebrates (Carletti et al., 2007; LaLone et al., 2012; Haider and Rauf, 2014; Guiloski et al., 2015; Ribas et al., 2016). There have been studies involving dexamethasone exposure in fish species, but these only examined physiologic effects associated with the stress response rather than harmful effects (Li et al., 2008; Lutton and Callard, 2008; Pierce et al., 2009). Hematological indices are important markers that reflect the health status of fish (Thummabancha et al., 2016). Based on the results of this study, the dexamethasone at higher concentration exerts hematologic changes including decreased RBC’s, hemoglobin, and MCHC. Consistent with our results, (Ribas et al., 2016) observed that in *Hopliasmala baricus*, the red blood cell count and hemoglobin level of fish exposed to dexamethasone decreased significantly. Also, Petrillo et al. (2017) stated that, as a result of dexamethasone treatment, erythrocytes, and hemoglobin levels decreased, while MCV was increased, suggesting a slight macrocytic anemia in pacu (*Piaractus mesopotamicus*). This anemia may be as a result of hematopoietic tissue damage, RBC’s are lysed and/or erythropoiesis is suppressed, causing this anemia. Cell membranes are likely to be more mechanically fragile as a result of this (Rosenberg et al., 1998), which was indicated by RBC’s alterations (data under preparation). Also, a reduction in RBC’s may also be attributed to hemodilution; nevertheless, they can result from the destruction of red blood cells or inability to synthesize new ones, resulting in reduced oxygen uptake by the cells (Dethloff et al., 2001).

The hematometric indices, MCV and MCH, were increased significantly only in the group exposed to low dose of dexamethasone. The MCHC show a marked decrease in levels within the high dexamethasone exposed group. As a result, these results are in agreement with that of Ribas et al. (2016), who observed an increase in these hematological parameters in *H. malabaricus* exposed to dexamethasone.

Fish have an adaptive immune response that relies heavily on lymphocytes; so a decrease in large lymphocytes represents a decrease in immunity (Ribas et al., 2016). No changes were observed in white blood cells count in fish exposed to lower and higher doses of dexamethasone. These results are in accordance with Reidarson and McBain (1999) as there was no change in leukocyte number in two Atlantic bottlenose dolphin after exposed to dexamethasone, and opposite to Guiloski et al. (2015) who exposed African catfish to dexamethasone, and observed an immunosuppressive state was manifested by a reduction of leukocyte counts. Such failure can render an organism more susceptible to infection (Ribas et al., 2016). Our study showed that dexamethasone high concentration exert immunological dysfunction in fish, which weakens its immune system and may lead to severe physiological disorders; the results reveal a significant changes in values including neutrophilia, monocytosois, and lymphocytopenia in large lymphocytes at 3.0 mg/L, and these results are in agreement with results of Davis et al. (2008).

Fish and their environments are assessed by biochemical parameters, such as glucose and proteins, which indicate the fish’s health status. Inappropriate carbohydrate metabolism is indicated by elevated blood glucose levels (Ramesh et al., 2018) and additionally, it may be used to detect fish stress due to environmental factors. In addition, changes in plasma protein levels indicate a person’s general health status and how toxicants affect their metabolism (Lavanya et al., 2011). Our results indicated an increase in serum glucose (hyperglycemia) levels in *Clarias gariepinus* exposed to different dexamethasone concentrations for 7 days. These results are in agreement with that of Fazio (2019) who observed a significant increase in serum glucose levels on sea bream (*Sparus aurata*) after exposure to stress. Similar results were reported in bottlenose dolphins (Reidarson and McBain). The significant higher glucose associated with the higher cortisol level was observed in the exposed groups. Cortisol levels increase during stress, and high cortisol levels increase gluconeogenesis and blood glucose (Vijayan et al., 2010).

Fish’s nutritional and immune statuses are affected by circulating proteins, such as albumin (Lingaiah et al., 2012). Our results indicated a significant decrease in albumin (hypoalbuminemia) and globulin content in *C. gariepinus* exposed to dexamethasone. These results are opposite to the findings of La Mear et al. (1997) who demonstrated that dexamethasone increased albumin and globulin in neonatal rats. In agreement with Haider and Rauf (2014), they reported that after exposure to sub-lethal doses of diazinon, the albumin and globulin contents of *Cirrhinus mrigala* significantly decreased.

Exposure to stress can affect kidney function. Studies indicate that stress can cause adverse changes in urination at multiple levels, not just phenomenologically (Shimizu et al., 2021). A decrease in kidney excretion and an excess of purine precursors during synthesis may cause high uric acid concentrations (Schmidt et al., 2013). The high levels of creatinine and urea in the blood are an indication of impaired kidney function, which is linked to ROS production and kidney damage (Upasani and Balaraman, 2003). Thus, this could explain the increase in the creatinine, urea, and uric acid levels in catfish serum subjected to high concentration of dexamethasone in the present study.

A measure of liver function is AST and ALT, which are aminotransferase enzymes. As long as these enzymes remain low during normal liver function, their increase indicates that the liver is not functioning normally and that it is impaired (Zhang et al., 2019). Our results indicated a marked increase in liver enzyme activity in *C. gariepinus* exposed to high dexamethasone concentration for 7 days. These findings are in agreement with that of Jerez-Cepa et al. (2019) who observed a significant increase in the activities of aspartate aminotransferase (AST) in gilthead sea bream (*Sparus aurata* L.) after exposure to dexamethasone and with Thomas et al. (2014) on bottlenose dolphins (*Tursiops truncatus*). These enzymes are considered indicators of damage to cell membranes when they are liberated from the cell and increase in blood levels (Banaee et al., 2014). These enzymatic changes may occur because of the damage of hepatocytes, which are responsible for detoxication and their accompanying exodus into the blood. In contrast, Yahi et al. (2017) observed that, dexamethasone treatment decreased liver enzyme levels, indicating that higher concentrations have hepatoprotective properties.

Cortisol is the most common stress indicator, and high levels of stress on fish induce elevation of cortisol levels (Martínez-Porchas et al., 2009). In the present study, the level of cortisol significantly increased in the exposed group to high dexamethasone and this result were in accordance with that of Zanuzzo et al. (2019) on *Piaractusm esopotamicus* and Jerez-Cepa et al. (2019) on *Sparus aurata*.

Inflammatory and immune responses are regulated by cytokines, which are water-soluble proteins. The fish immune status can be enhanced by supplementing their diet with immuno-stimulants to stimulate the production of inflammatory cytokines (Awad et al., 2011). It has been shown that leukocytes produce the majority of inflammation cytokines, including IL-*1β*, and these play an important role in the host’s defense against infection, necrosis, and other forms of inflammation (Myren, 2007).

Pleiotropic cytokines regulate immune responses, hematopoiesis, inflammation, and oncogenesis through a variety of biological functions. In the present study, there was a significant increase in IL-*1*β and IL-6 production in serum and our findings agree with Begam and Sengupta (2015) who noted an increase in proinflamatory cytokines the in Channa punctatus after exposure to mercury.

Aquatic organisms were tested for various environmental stress effects using antioxidant biomarkers (Sayed et al., 2021). In the present study, the activities of SOD and TAC were significantly decreased in the two groups of exposure, these results agree with Hamed et al. (2020) as they reported a decrease in total antioxidant capacity (TAC) in Nile Tilapia after exposure to microplastic and with disagreement with Hoseini et al. (2022) as they noted a significant increase in SOD levels in common carp as a result of stress, whereas malondialdehyde (MDA) concentration showed a significant increase in the low dose and a significant increase in the high dose dexamethasone exposed group. These results are in accordance with Carneiro et al. (2022). In contrast, the levels of antioxidant enzymes were decreased compared with the control. Sayed et al. (2021) reported that there was a depletion of antioxidant enzymes in catfish (*Clarias gariepinus*) after exposure to hydroxychloroquine. In contrast, José et al. (1997) observed an increase in catalase and superoxide dismutase enzymes in the adult rat lung after dexamethasone injection. Also, calves orally treated with dexamethasone showed a significant increase of both glutathione peroxidase isoforms and SAC (Carletti et al., 2007). In organisms with a nervous system and muscle tissue, acetylcholinesterase (AChE) is a serine hydrolase enzyme. In aquatic ecosystems, it has been widely used as a biomarker for detecting neurotoxicity of pollutants that are involved in neurotransmission (Jebali et al., 2013; Kim and Lee, 2018). Studies have indicated that AChE concentration is inhibited in aquatic environments by environmental such as pharmaceuticals (Piner and Üner, 2012; Rhee et al., 2013). Data from our research revealed a marked decrease in AChE concentration in blood serum with the increase of dexamethasone concentrations to the groups after 7 days of exposure, our result matched with Brank et al. (1998) who observed a reduction in AchE activity in rat skeletal muscle after dexamethasone exposure. Also, Al-Ghais (2013) noticed that decrease in AChE concentration in *Tilapia mossambica* (Peters) reared in sewage treatment plant (STP) effluently.

In conclusion, high consumption of drugs such as dexamethasone, combined with their presence in the environment, increases concerns about its consequences and negative impacts in aquatic organisms, for example, fish. Hence, dexamethasone at concentrations present in environmental water sources may cause deleterious effects on blood biomarkers, biochemical, and antioxidant factors as well as an upregulation in the immune response of catfish even after the depuration period.

## Data Availability

The raw data supporting the conclusions of this article will be made available by the authors, without undue reservation.
